# Preprocessing of 2-Dimensional Gel Electrophoresis Images Applied to Proteomic Analysis: A Review

**DOI:** 10.1016/j.gpb.2017.10.001

**Published:** 2018-02-21

**Authors:** Manuel Mauricio Goez, Maria Constanza Torres-Madroñero, Sarah Röthlisberger, Edilson Delgado-Trejos

**Affiliations:** 1 Automatics, Electronics and Computer Science Research Group, Faculty of Engineering, Instituto Tecnologico Metropolitano, Medellin 050012, Colombia; 2 Biomedical Innovation and Research Group, Faculty of Applied and Exact Sciences, Instituto Tecnologico Metropolitano, Medellin 050012, Colombia; 3 Quality, Metrology and Production Research Group, Faculty of Economic and Management Sciences, Instituto Tecnologico Metropolitano, Medellin 050012, Colombia

**Keywords:** Background correction, Filtering, Noise reduction, Preprocessing, 2D gel electrophoresis

## Abstract

Various methods and specialized software programs are available for processing two-dimensional gel electrophoresis (2-DGE) images. However, due to the anomalies present in these images, a reliable, automated, and highly reproducible system for 2-DGE image analysis has still not been achieved. The most common anomalies found in 2-DGE images include vertical and horizontal streaking, fuzzy spots, and background noise, which greatly complicate computational analysis. In this paper, we review the **preprocessing** techniques applied to 2-DGE images for **noise reduction**, intensity normalization, and **background correction**. We also present a quantitative comparison of non-linear **filtering** techniques applied to synthetic gel images, through analyzing the performance of the filters under specific conditions. Synthetic proteins were modeled into a two-dimensional Gaussian distribution with adjustable parameters for changing the size, intensity, and degradation. Three types of noise were added to the images: Gaussian, Rayleigh, and exponential, with signal-to-noise ratios (SNRs) ranging 8–20 decibels (dB). We compared the performance of wavelet, contourlet, total variation (TV), and wavelet-total variation (WTTV) techniques using parameters SNR and spot efficiency. In terms of spot efficiency, contourlet and TV were more sensitive to noise than wavelet and WTTV. Wavelet worked the best for images with SNR ranging 10–20 dB, whereas WTTV performed better with high noise levels. Wavelet also presented the best performance with any level of Gaussian noise and low levels (20–14 dB) of Rayleigh and exponential noise in terms of SNR. Finally, the performance of the non-linear filtering techniques was evaluated using a real 2-DGE image with previously identified proteins marked. Wavelet achieved the best detection rate for the real image.

## Introduction

Proteomics is the analysis of the complete set of proteins (*i.e.*, the proteome) produced in a cell, tissue, or organism, at a given time. A number of different aspects of protein analysis are covered in proteomics, including the analysis of protein expression. The presence or absence of proteins, and the direct measurement of relative protein abundances can help understand cellular processes, and may be useful for identifying drug targets and diagnostic/prognostic markers [1]. As such, it is one of the most active fields of biological research, given its wide range of applications.

One of the most commonly used techniques in comparative proteomic studies is two-dimensional gel electrophoresis (2-DGE) [2], a technique capable of resolving thousands of proteins in a single run. Using 2-DGE, proteins are firstly separated according to their isoelectric points (pIs) in one dimension, and then, according to their molecular weights in a second dimension [3]. Afterward, the 2-DGE gels are stained for protein visualization and analyzed with computer-assisted image evaluation systems for a comprehensive qualitative and quantitative examination of the proteomes [4].

2-DGE was initially employed for protein separation and analysis approximately in 1975 [5]. Even though it was introduced more than four decades ago, nowadays 2-DGE is still widely used for whole proteome analysis [6], [7], comparative analysis of proteome changes [6], [8], biomarker discovery, cancer research [9], [10], as well as for the identification of protein isoforms and post-translational modifications [11], among other purposes. Its popularity could be attributed to several factors. It is possible to resolve and visualize thousands of proteins in a single gel as mentioned above. In addition, 2DGE is very affordable compared to other techniques, which does not require complex laboratory equipment but offers reliable and reproducible results. 2GDE is optimal for cases when multiple analyses are required on replicate samples. More importantly, it is compatible with mass spectrometry and other downstream analyses. Therefore, 2GDE is often used as one of the first steps in describing the protein composition of a particular sample type, at a certain time point, and under a particular set of conditions.

Many computational applications are available for processing and analyzing 2-DGE images, such as MELANIE, PDQuest, Z3, Progenesis Workstation, ProteomeWeaver, ProteinMine, Delta2D, and DeCyder [12], [13]. Given that one cell can express around ten thousand proteins, 2-DGE needs an effective computational tool that can process large volumes of information [14]. It is often necessary to apply appropriate preprocessing techniques to 2-DGE images, as the ultimate performance of these analysis tools strongly depends on the quality of the images to be processed [4], [15].

For 2-DGE image analysis, techniques are required to detect protein spots, to segment and to quantify the protein expression level based on the number of pixels [15], [16]. An additional step of image alignment is performed in order to match the corresponding protein spots from different images [17]. However, due to technical difficulties inherent to 2-DGE, anomalies are often found in gel images, such as noise around protein spots, vertical and horizontal streaking, saturation of certain protein spots, presence of very faint protein spots, as well as non-linear intensity of protein spots [14], [18]. Therefore, 2-DGE image analysis is frequently perturbed by the presence of different types and levels of noise. For instance, the impulsive noise can spectrally interfere in all frequencies when the sample is digitalized. The background of 2-DGE images can also vary among samples, depending on the technical specifications of the imaging system used to capture and digitalize the images [15]. These variations and anomalies often complicate the analysis of 2-DGE images and affect the reproducibility of the results obtained [19], [20]. A preprocessing phase could be effective in reducing or eliminating these anomalies, thus reducing errors in the subsequent spot detection. Omitting this preprocessing stage may profoundly affect the results of the subsequent analysis, as noise could be falsely identified as a protein, whereas real proteins of interest could be missed [21]. Therefore, it is important to review the advances in 2-DGE image preprocessing, in order to identify areas of improvement that may be of great interest to researchers in the field.

There are three common objectives for the 2-DGE image preprocessing, *i.e.*, pixel intensity regularization (image normalization), background correction, and noise reduction (filtration) [15]. In the subsequent sections, we describe in detail the preprocessing strategies to achieve these objectives, their limitations, and recent advances. We also compare the most representative noise reduction techniques using synthetic gel images for a quantitative evaluation. Finally, we conclude with recommendations to be considered for successful automatic 2-DGE image processing.

## Preprocessing of 2-DGE images

**Figure 1:**
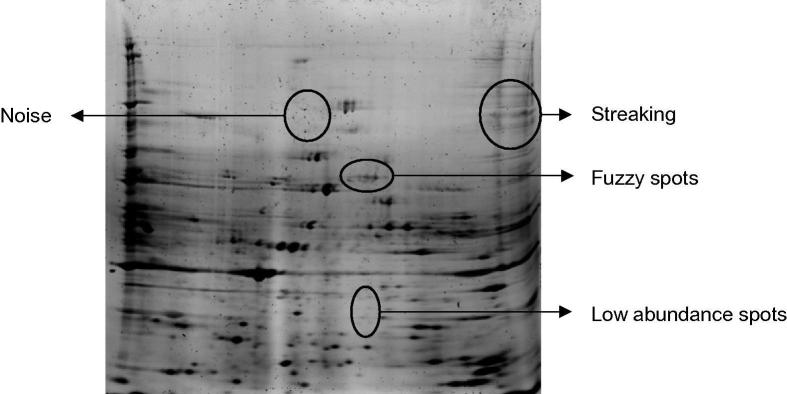
**Common anomalies present in a 2-DGE image** A 2-DGE image obtained from human peripheral blood mononuclear cells. As indicated by the arrows, the most common anomalies are vertical and horizontal streaking, fuzzy spots, faint (low abundance) spots, and background noise. 2-DGE, 2-dimensional gel electrophoresis.

2-DGE images inevitably exhibit anomalies caused by sample preparation techniques and the imaging system used to acquire the digital images [22]. The most common anomalies in 2-DGE images are oversaturated, faint, or fuzzy spots, vertical and horizontal streaking, overlapping spots, and noise [13], [23]. Figure 1 shows a 2-DGE image with the most common anomalies. The image was obtained in our laboratory from prostate tissue, the challenges of image preprocessing, and thus, the requirement for robust algorithms in order to achieve proper preprocessing are apparent.

### Image normalization

Intensity normalization of 2-DGE images aims to normalize the intensity of protein spots to facilitate processing. The spot intensities may vary due to differences in the exposure of the gel during the staining process and data saturation, among other reasons [15]. As a step of image processing [24], intensity normalization is also known as a preprocessing technique in various literatures [25]. In fact, some researchers recommend eliminating saturated spots before processing in order to achieve the optimal results for the identification of proteins in the gel [14], [26].

To avoid saturation caused by high protein concentrations, it is necessary to make sure that most of the data are represented by levels slightly below the maximum possible intensity. Highly abundant proteins often mask fainter spots that represent proteins with low abundance. Usually, these low-abundance proteins are also relevant, but pose difficulties in accurate quantification, if the spots are too faint [27].

The normalization process helps to reduce noise, which distorts the detection of protein spots in 2-DGE images according to criteria of statistical significance, thus allowing the outline of the spots of interest to be delineated [28]. However, in some circumstances, the intensity of pixels does not present a clear tendency. This type of standardization is based on information from various replicate gels, generating a statistical average of the intensity of each pixel. With these statistical averages, the intensities of the entire image are normalized, using normalization algorithms such as Cyclic Loess, Contrast, Cyclic Linear, and Quantile [28]. Image regularization with multiple replicate gels has yielded satisfactory results, since changes in patterns of protein expression among samples are small compared to the overall pattern of protein spots. The major limitation of this approach lies in obtaining and aligning a representative number of gel images, which may result in the unwanted removal of faint protein spots [15]. With image regularization, it is possible to minimize distortions present in the images; however, low-abundance proteins are often eliminated in the process. Therefore, it is necessary to apply discriminatory algorithms to these regularization techniques, so that potentially faint but important protein spots would not be removed during image regularization.

### Background correction

The aim of 2-DGE image processing is to detect spots, and define the location, border, and intensity of each spot. However, in some conditions, defining the limits of the spot border is difficult due to the varied background, noise, and saturation of protein spots [14], [29], [30]. Background correction is thus necessary, as the background signal of 2-DGE images is not uniform and varies locally according to protein intensities. The greater the intensity of a protein spot, the greater the noise signal around it, which introduces errors in detection. In addition, anomalies such as vertical streaking must be removed from the image, as protein spots contained within these streaks are not identifiable or quantifiable [15], [31]. Three background correction techniques are available to reduce the influences of these phenomena. These include adjustment by polynomials, local and global minima, and histograms.

Background correction with polynomial adjustment has been widely accepted in the scientific community [32]. This technique seeks smoothing the image background, removing noise from overlapping spots, and minimizing the effects of intensity degradation. The biggest limitation of background correction is the selection of a background sample for the adjustment. A poor selection of the background sample can generate significant image distortion, or even lead to the improper softening of background texture [33]. Local and global minima are the simplest technique for background correction, making it possible to effectively mitigate noise in the flatter areas (without significant changes in pixels) and smooth discontinuities. However, a global threshold is not sufficient to detect background variations [15].

For background adjustment using histograms, the distribution of intensities in an image is used to identify the background. The maximum peak in the intensity distribution is the intensity of background pixels. The challenge of this technique is to establish the intensity threshold of the background that is usually set by an optimization process [34]. The selection of parameters is a significant drawback for users with little knowledge of optimization techniques, considering that the model implemented to describe the background may be insufficient or cause a significant loss of faint but important protein spots [35]. Although studies on the implementation of background correction techniques for 2-DGE images have lost momentum, a variety of techniques [36] in this field are yet to be explored. In addition, new techniques have also been developed that are efficient for the analysis of images with features similar to those of 2-DGE images, such as combination of Gaussian mixture models and machine learning approach [37], which may be promising options for 2-DGE image processing.

### Noise reduction

2-DGE image filtering is the most commonly used approach within the scientific community, with the most diversified techniques [15]. These filtering techniques can be classified into two broad categories: linear and nonlinear filters.

The first techniques reported for 2-DGE image’ preprocessing were based on linear filters, which are the most widely used techniques in commercial software programs. These kinds of filters compute the output pixel as a linear relation of the surrounding pixels [38]. In general, linear filters tend to blur images, affecting the edges, lines, and fine details of the protein spots [39]. While linear filtering techniques, such as the Gaussian filters, have an acceptable performance under general conditions, they are also likely to reduce the density of proteins spots by smoothing the contour [40].

Orthogonal regression methods have been used as a simple and efficient option that can adapt to a linear model [41]. However, the edges of protein spots of interest in 2-DGE images are often distorted during filtering [25], [42]. The linear filters based on orthogonal regression techniques, which are most commonly used for noise reduction in 2-DGE images, are based on principal component analysis (PCA) and Wiener filters. PCA is a robust orthogonal regression technique, which shows a great advantage in terms of spot edge maintenance. However, small details, such as low-abundance protein spots, can be removed in the process [43], [44].

With the Wiener filter, it is possible to obtain satisfactory results with a low level of distortion of the image, but the noise reduction is compromised [38]. When taking into account the overall advantages of noise removal, a certain level of signal degradation is generally acceptable. However, the noise reduction results in the blurred image with poor edge definition [15]. In general, these methods allow the user to select the number of times the smoothing filter is applied, and the size of the convolution window to adjust the level of smoothing [45].

Nonlinear filters have fewer limitations, but generally increase the degree of complexity and the need to define parameters more thoroughly. Some nonlinear filtering techniques used in 2-DGE images include contourlet transform [46], total variation [47], and wavelet transform [19]. The contourlet transform performs a decomposition of the data in frequency and space by means of two decomposition methods [46]. These methods allow a multiple scale of space and frequency, as well as a high degree of directionality. Satisfactory results have been achieved using these methods, in addressing the low signal-to-noise ratio [48], and loss of relevant information, thereby surpassing the wavelet transform [48].

The total variation technique, proposed by Chan [49] and Xin [50], is very advantageous in terms of the extraction of geometric features and in terms of the preservation of edges. Using this technique, different degrees of smoothing on different points can be achieved, which exemplifies its anisotropy. Although able to capture all the directional information, this technique leads to staircase effects in the noise removal process [51]. Therefore, this technique does not perform as well as the wavelet transform technique [41], [50]. Wavelet transform is the most commonly used technique for noise reduction, with a high percentage of filtering and acceptable level of loss of relevant information [19], [43], [52], [53], [54], [55], [56], [57]. This method has become increasingly popular in the preprocessing of 2-DGE images since 2004, as reported in the study by Kaczmarek and others [19].

Despite the simplicity of noise suppression methods based on spatial filtering, the image tends to be severely distorted after processing. As these types of images have local features with high variation, it is almost impossible to discriminate between information of interest and noise, when the processing considered only the spatial or spectral domain [41]. For this reason, it is necessary to use a filter with a joint time–frequency domain [57] that exceeds spatial filtering, in both signal/noise ratio and distortion. Using the wavelet transform method, the image can be decomposed into space-frequency components and evaluated point by point. It is also possible to combine several images in order to obtain an average value and thus eliminate noise more effectively [56]. For a correct operation, it is necessary to determine the scale of the wavelet transform. However, it is difficult to properly define the contours, resulting in a significant distortion of the information of interest [50].

Different derivations of the wavelet transform [58], [59] are available to deal with its limitations. For instance, Barlaud and colleagues [60] presented a very useful technique called Wavelet Transform Quincunx, to obtain a multiresolution scale, thereby improving the performance around the edges and enhancing filtering effect [41]. A hybrid technique, WTTV, combines the power of wavelets with the advantages of Total Variation (TV) [50], to reduce the loss of relevant information and increases the level of filtering. The wavelet transform can be applied first, followed by a total variation routine, thus minimizing distortions caused by the wavelet transform. In this case, it is necessary to carefully define the parameters for both components, since false information is generally introduced due to limitations of the directional information. As a result, the geometric captures can be affected, creating a distortion of the borders around the spots of interest [46].

Due to the limitations of these techniques and the need for parameter adjustments, advanced techniques such as genetic algorithms [52] have been applied. However, it is noteworthy that this entails an increase in computational complexity. Although methods are available to minimize the computational cost of genetic algorithms, these add a higher level of complexity to the whole process [61].

### Validation measures for processing 2-DGE images

The lack of performance indicators with which the various techniques can be fairly compared greatly hinders the selection of preprocessing techniques. In fact, performance comparison of many commonly used techniques has not yet been reported in the literature [15]. The most commonly used indicator is the signal-to-noise ratio (SNR) obtained from the mean square error (MSE):

(1) MSEn=∑i=1n(xi-x^i)2∑i=1n(xi)2

(2) $$\text{SNR}=10*\log_{10}\frac{1}{{\text{MSE}}_{n}}$$

However, with the SNR indicator, important factors regarding the retention of relevant information are not considered. Daszykowski thus proposed a measure called false discovery rate (FDR), defined as a ratio of the number of stable variables from the permuted data to the number of stable variables from the original dataset [25]. While this measure is focused on the object detection process, it does not list the spots of interest that were removed during the preprocessing routine. FDR can be estimated by:

(3) $$\text{FDR}=100*\frac{N1}{N2}$$

where N1 is the number of falsely identified significant features in the permuted data for a given threshold value and N2 is the number of significant features in the experimental data set for the same threshold.

Another measure is spot efficiency [41] as defined below, which establishes a relationship between the false spots that were generated or prevailed, and spots that were lost with the technique.

(4) $$\text{Spot efficiency}=\frac{\text{Number of spots detected}-\text{Number of false spots}}{\text{Number of spots detected}+\text{Number of lost spots}}$$

## Quantitative comparison of noise reduction methods

### Comparison of synthetic images

**Figure 2:**
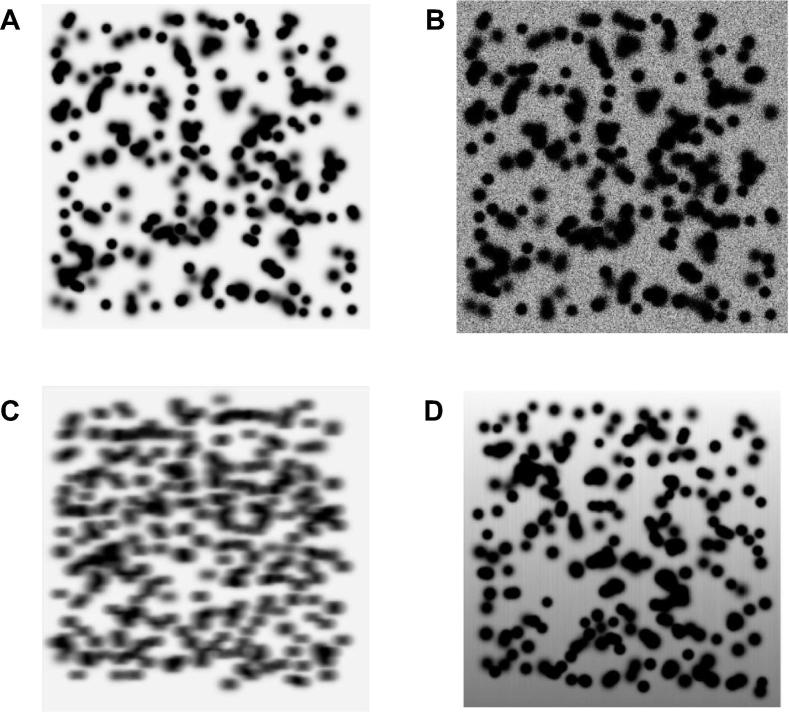
**Synthetic protein spots modeled as a 2-D Gaussian distribution** **A.** Example of synthetic image. **B.** Synthetic image with noise obtained by adding random values with Gaussian distribution. **C.** Synthetic image with blurring using a filter of displacement of 5 pixels. **D.** Synthetic image with distortion that change background intensity until 5%.

The performance of several noise reduction techniques was compared using synthetic images. Experiments were developed under controlled conditions and the quantitative indicators SNR and spot efficiency were used to determine the performance of the filtering techniques with a perturbation of impulsive-type noise. To generate a cloud of spots, we used a model of uniform distribution, which simulates proteins in 2-D gels, and allowed overlapping between spots. The synthetic spot model is a Gaussian distribution [62], with adjustable parameters for size, intensity, and degradation (see Figure 2A). We used a 512 × 512 synthetic image with 250 protein spots. For each protein, the standard deviation was randomly set between 0.3 and 0.8, and its maximum intensity was constrained between 0.6 and 1. Different types of noise were added to the synthetic images to examine how the change of distribution affects the behavior of the techniques being evaluated. The noise distributions applied to the images are: Gaussian noise, Rayleigh type noise, and exponential type noise [63]. Table 1 presents the parameters used for each noise type to simulate images with SNR between 8 and 20 dB. An example of a synthetic image with noise is presented in Figure 2B.

**Table 1:** **Parameters for Gaussian, Rayleigh and exponentialnoise of synthetic images**

**Distribution**	**Parameter**	**Noise intensity (dB)**						
		**20**	**18**	**16**	**14**	**12**	**10**	**8**
Gaussian	*σ*	0.0766	0.0966	0.1214	0.1530	0.1929	0.2430	0.3091
Rayleigh	*b*	0.0277	0.0398	0.0539	0.0725	0.0980	0.1335	0.1858
Exponential	*a*	14.0361	10.0361	8.3389	6.8749	5.6862	4.6894	3.8549

*Note*: *σ*, standard deviation for Gaussian distribution with median *μ* = 0; *b*, scale for Rayleigh distribution with shifting parameter *α* = 0; *a*, scale parameter for exponential distribution.

To obtain images with a blurring effect, a linear approximation of the camera movements in pixels (*N*) was performed, using image processing techniques with a filter of displacement vector [64]. We used synthetic images with linear displacement from 5 to 30 pixels and a representative synthetic image with blurring effect is shown in Figure 2C. In addition, we also generated images with distortions between 5% and 30% in the background to represent a non-uniform illumination system, as exemplified by the image in Figure 2D.

The SNR and spot efficiency for the images with noise and filtered with nonlinear techniques wavelet, contourlet, TV, and WVTV are presented in Table 2 and Table 3, respectively. The technique with the best performance for each noise distribution, variance, and indicator is highlighted. As expected, the performance of each of these techniques decreases with the increasing noise level. In terms of SNR, wavelet is the best technique for images with Gaussian noise (Table 2). For images with Rayleigh and exponential noise, wavelet presents the best performance for low-level noise. Conversely, TV was better for high level of Rayleigh and exponential noise (8–12 dB).

**Table 2:** **Performance of noise reduction techniques evaluatedusing SNR**

**Noise type**	**Noise reduction technique**	**Noise intensity (dB)**						
		**20**	**18**	**16**	**14**	**12**	**10**	**8**
Gaussian	Wavelet	**27.61**	**26.70**	**25.55**	**24.24**	**22.82**	**24.15**	**19.29**
	Contourlet	25.80	24.08	22.36	20.79	19.41	18.26	17.33
	TV	26.29	24.48	22.64	20.79	18.92	16.99	15.02
	WVTV	20.01	19.97	19.90	18.76	18.75	18.63	18.46

Rayleigh	Wavelet	**27.54**	**24.76**	**21.04**	**17.77**	14.95	12.35	9.86
	Contourlet	25.75	20.03	19.90	16.97	14.42	11.96	9.25
	TV	26.11	23.87	20.73	17.69	**15.01**	**12.50**	**10.50**
	WVTV	20.30	19.59	18.15	16.20	14.12	11.73	8.78

Exponential	Wavelet	**25.15**	**6.69**	**24.63**	21.38	18.30	15.41	12.72
	Contourlet	23.63	22.95	20.84	18.35	16.05	13.76	11.58
	TV	23.53	24.78	24.20	**22.28**	**19.86**	**17.20**	**14.60**
	WVTV	18.28	18.71	18.47	17.60	16.22	14.37	12.29

*Note*: SNR, signal-to-noise ratio; TV, total variation; WVTV, wavelet-total variation. The values in bold indicate the best SNR for each noise level.

**Table 3:** **Performance of noise reduction techniques evaluatedusing spot efficiency**

**Noise type**	**Noise reduction technique**	**Noise intensity (dB)**						
		**20**	**18**	**16**	**14**	**12**	**10**	**8**
Gaussian	Wavelet	**90.71**	**90.00**	**90.36**	**87.86**	**88.57**	86.07	71.79
	Contourlet	89.29	88.57	87.50	87.51	78.93	65.00	44.64
	TV	87.14	89.64	86.79	85.00	72.21	37.89	25.12
	WVTV	87.14	86.43	86.43	85.71	86.79	**87.14**	**85.16**

Rayleigh	Wavelet	**90.36**	**89.64**	**89.64**	**91.43**	**89.64**	**87.86**	**88.57**
	Contourlet	89.64	88.57	88.57	86.07	89.29	84.64	72.36
	TV	87.86	87.86	85.36	88.57	85.43	87.59	51.25
	WVTV	86.43	86.79	87.86	85.00	86.79	86.07	83.57

Exponential	Wavelet	90.00	**90.00**	**88.93**	**88.21**	**88.21**	**88.21**	77.14
	Contourlet	90.36	87.14	87.86	84.29	72.50	61.84	48.25
	TV	**90.71**	89.29	87.50	86.07	82.86	75.00	62.37
	WVTV	85.71	85.36	85.36	85.71	84.29	85.57	**83.93**

*Note*: TV, total variation; WVTV, wavelet-total variation. The values in bold indicate the best spot efficiency for each noise level.

**Figure 3:**
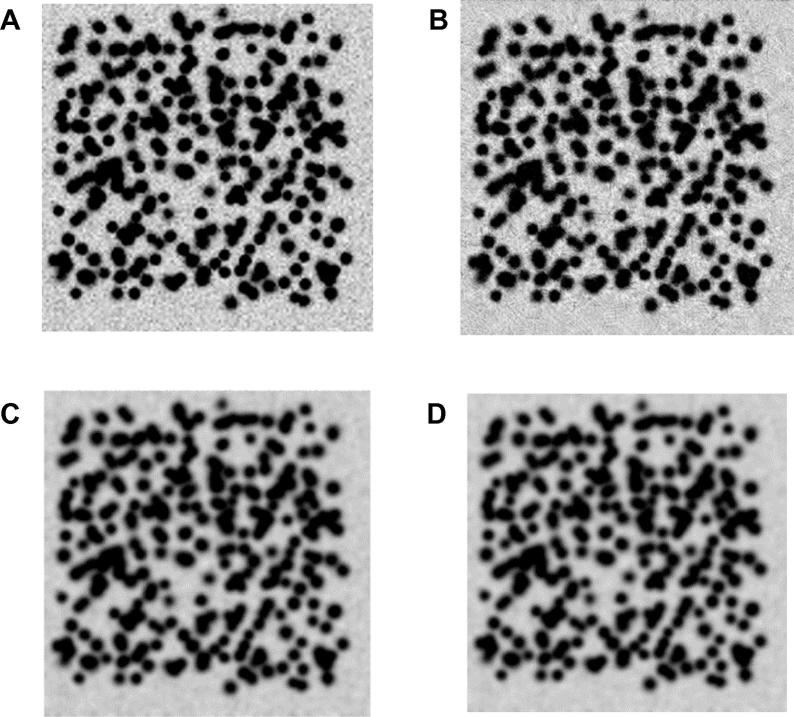
**Filtering results for a 2-DGE synthetic image with Gaussian noise** **A.** Synthetic image filtered with wavelet. **B.** Synthetic image filtered with contourlet. **C.** Synthetic image filtered with TV. **D.** Synthetic image filtered with WVTV. The synthetic image is obtained by adding random values with Gaussian distribution for an intensity of the noise of 10 dB. TV, total variation; WVTV, wavelet-total variation.

In terms of spot efficiency, wavelet and WVTV techniques have a lower sensitivity to noise than the contourlet and TV techniques, as reflected by the best performance of wavelet and WTTV techniques. Wavelet was the best for images with noise levels between 12 and 20 dB, and WVTV was best for images with high-level noise. Figure 3 presents an example of filtering results obtained for the image with Gaussian noise equal to 10 dB.

Table 4 presents SNR and spot efficiency for images with the blurring effect and non-uniform background distribution filtered with nonlinear techniques wavelet, contourlet, TV, and WVTV. Contourlet and wavelet transform have the best performance with blurred images and images with a non-uniform background distribution. Both filtering techniques achieved comparable results for the different levels of blurring and distortion.

**Table 4:** **Performance of noise reduction techniques evaluated using SNR and spot efficiency for images with blurring and non-uniformbackground distribution**

**Image anomaly**	**Noise reduction technique**	**SNR (dB)**						**Spot efficiency (%)**					
Blurring	*N* (pixels)	5	10	15	20	25	30	5	10	15	20	25	30
	Wavelet	34.8	**26.2**	**21.6**	**18.4**	**16.1**	**14.3**	**98**	**96**	**90**	80	**66**	54
	Contourlet	**35.3**	**26.3**	**21.6**	**18.4**	**16.1**	**14.3**	**98**	**96**	**90**	81	65	53
	TV	22.6	22.2	20.6	17.9	16.8	14.2	91	92	89	**82**	63	54
	WVTV	25.4	24.3	20.7	17.9	15.9	14.2	93	94	89	82	63	**55**

Non-uniform background	Distortion (%)	5	10	15	20	25	30	5	10	15	20	25	30
	Wavelet	**21.5**	**19.8**	**17.9**	**15.7**	**12.9**	**9.2**	**99**	98	**99**	97	97	**97**
	Contourlet	**21.5**	**19.8**	**17.9**	**15.7**	**12.9**	**9.2**	**99**	**99**	**99**	**98**	**98**	96
	TV	19.3	18.3	16.9	15.1	16.7	9.1	92	92	92	92	94	94
	WVTV	20.3	19.0	17.4	15.4	12.8	9.1	98	97	98	97	96	96

*Note*: The values in bold indicate the best SNR and spot efficiency for each noise level. *N* (pixels) represents the number of pixels used to simulate blurring effect. Distortion (%) is the percentage of degradation used for the non-uniform background.

### Comparison with a real 2-DGE image

**Figure 4:**
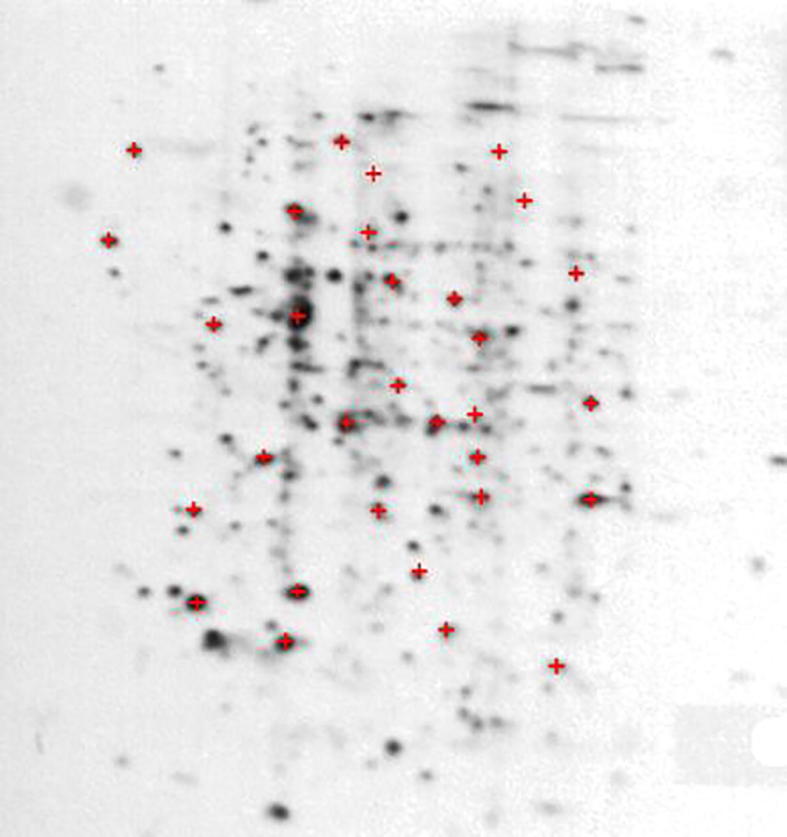
**An annotated 2-DGE image of a human leukemia blood sample** A 2-DGE image of a human leukemia blood sample [65] was used to validate the filtering results obtained from the synthetic images. Annotated proteins in the image are indicated with crosshairs.

A real 2-DGE image taken from the LECB 2-D PAGE Gel Images Datasets was used to compare the performance of the aforementioned filtering techniques [65]. Sample 19, an annotated 2-DGE image of human leukemia (Figure 4), in which many proteins have been previously identified was selected for computing the rate of detection of these known proteins before and after filtering using the different techniques. Table 5 presents the rate of detected proteins from the original image, and of the image filtered by wavelet, contourlet, TV, and WVTV. It can be seen that wavelet, contourlet, and WVTV improved the rate of detection in comparison with the non-filtered image. A detection rate of 71.2% was achieved using TV, whereas a maximum detection rate of 87.9% was achieved using wavelet.

**Table 5:** **Performance of noise reduction techniques evaluated using the rate of detected proteins in areal 2-DGE image**

**Image**	**Detection rate**
Original	75.8%
Filtered by wavelet	**87.9%**
Filtered by contourlet	81.8%
Filtered by TV	71.2%
Filtered by WVTV	78.8%

*Note*: The value in bold indicates the best detection rate achieved. Data source: LECB 2-D PAGE Gel Images Dataset.

## Remarks

The first step in most commercial 2-DGE image analysis software programs is spot detection. Often, false positive spots are detected due to noise and other artifacts in the image, and conversely, some real data may be missed. Therefore, image preprocessing is a fundamental step for proteomic analysis using 2-DGE, as the performance of the subsequent analysis is directly affected by the quality of the image. The development of image acquisition systems and optimal sample preparation protocols greatly improves image quality, reducing the intensity and frequency of unwanted anomalies. However, some anomalies are unavoidable and affect the accuracy and reproducibility of the proteomic characterization of the sample. Each of the preprocessing approaches meets particular needs. Nonetheless, the order these preprocessing stages should be implemented in, and whether all of them are actually necessary or some could be excluded without affecting the final performance, is still not clear. In conclusion, a methodology that adequately integrates these preprocessing approaches is needed.

Even though several specialized image processing software programs are available for 2-DGE, these still require considerable human intervention, which affects reproducibility of the results as well. The wide range of anomalies in 2-DGE images, such as fuzzy spots, vertical and horizontal streaking, and noise, make it difficult to process the image. Therefore, it is important to implement advanced preprocessing techniques that could help mitigate the effect of these anomalies. In this review, we grouped three approaches of preprocessing: filtering, image regularization, and background correction, which are suited to different purposes such as noise reduction, regularization of the intensity of the pixels, and background correction. If these preprocessing procedures are not implemented, negative effects are observed during the image processing stage, such as image distortion, loss of relevant information, and changes in the contour of the spots of interest, yielding non-representative data. Table 6 summarizes the most common techniques, and the disadvantages of the three main groups of approaches for 2-DGE image pre-processing.

**Table 6:** **Summary of preprocessing techniques for2-DGE images**

**Objective**	**Approach**	**Disadvantage**
Image normalization	Multiple-gel	Requiring alignment of samples

Background correction	Adjustment of polynomial	Requiring selection of background sample
	Local and global minima	Depending on threshold selection
	Adjustment using histogram	Requiring setting of optimization parameters

Filtering	Wavelet	Requiring scale selection
	Contourlet	Requiring scale selection
	TV	Resulting in the staircase effect
	WVTV	Depending on parameter of both wavelet and TV

Noise reduction is successful when filtering techniques are applied. Although linear filters are easy and simple for reducing noise in 2-DGE images, these filters tend to distort the edges of the spots of interest. Techniques such as Gaussian filters, orthogonal regression, and PCA often remove small details such as low-abundance proteins in the process. Thus, nonlinear noise reduction techniques are presented as more appropriate solutions. Nonlinear filters are more robust, but also more complex to use; they require the definition of parameters that depend on the image. It is clear that there is still a great scope for improving the performance of preprocessing techniques for 2-DGE images, as well as for proposing new ones, since the decision to implement these techniques in 2-DGE image processing is based on their performance with other types of images. Therefore, 2-DGE images are an attractive alternative for testing new algorithms for noise reduction, background correction, and image adjustment.

In this article, we validated the quantitative performance of the most representative preprocessing techniques for noise reduction of 2-DGE images. By comparing the performance of wavelet, contourlet, TV, and WVTV techniques using SNR and spot efficiency indicators, we conclude that spot efficiency is more appropriate for evaluating the noise reduction techniques applied to 2-DGE images. This index provides information about the effect of the noise reduction technique on the process of protein detection. Quantitative performance comparison using synthetic images indicates that wavelet filtering is the best technique under the test conditions, which achieves good results for the images with different types and levels of noise evaluated.

Taking into account the results obtained for the images with a blurring effect, and the fact that this anomaly is recurrent [66], we highlight the importance of high-quality image acquisition equipment, as this blurring effect is not mitigated effectively by the preprocessing techniques evaluated in this review. Blurring dramatically affects the performance of detection algorithms, and hence, specialized deblurring algorithms could be included in the preprocessing stage of 2-DGE images. However, cautions should be taken when including these techniques since they can lead to a significant distortion of the image [67].

## Competing interests

The authors have declared no competing interests.
